# Modelling Granular Process Flow Information to Reduce Bottlenecks in the Emergency Department

**DOI:** 10.3390/healthcare10050942

**Published:** 2022-05-19

**Authors:** Marian Amissah, Sudakshina Lahiri

**Affiliations:** Institute of Digital Healthcare, WMG, University of Warwick, Coventry CV4 7AL, UK; m.amissah@warwick.ac.uk

**Keywords:** emergency department, bottlenecks, waiting time, variation, quality, process modelling, crowding

## Abstract

Increasing demand and changing case-mix have resulted in bottlenecks and longer waiting times in emergency departments (ED). However, many process improvement efforts addressing the bottlenecks have limitations, as they lack accurate models of the real system as input accounting for operational complexities. To understand the limitations, this research modelled granular procedural information, to analyse processes in a Level-1 ED of a 1200-bed teaching hospital in the UK. Semi-structured interviews with 21 clinicians and direct observations provided the necessary information. Results identified Majors as the most crowded area, hence, a systems modelling technique, role activity diagram, was used to derive highly granular process maps illustrating care in Majors which were further validated by 6 additional clinicians. Bottlenecks observed in Majors included awaiting specialist input, tests outside the ED, awaiting transportation, bed search, and inpatient handover. Process mapping revealed opportunities for using precedence information to reduce repeat tests; informed alerting; and provisioning for operational complexity into ED processes as steps to potentially alleviate bottlenecks. Another result is that this is the first study to map care processes in Majors, the area within the ED that treats complex patients whose care journeys are susceptible to variations. Findings have implications on the development of improvement approaches for managing bottlenecks.

## 1. Introduction

Timely care is an important quality indicator (QI) in emergency departments (ED) [1,2,3,4,5,6,7,8]. At the same time, EDs are experiencing unprecedented rises in demand [5,9,10,11,12,13,14,15,16,17,18]. Moreover, the case-mix of the demand is also changing since, in addition to the critically ill, EDs are seeing more cases with chronic illnesses [19], medical patients with complex symptoms [20], as well as non-urgent presentations [21], putting additional pressures on resources and clinician time. At an operational level, this has resulted in bottlenecks [5,14,22] adding to longer waiting times [23] and inefficient care [24]. These factors are also associated with overcrowding in EDs, a problem that has raised global concern [3,6,15,25,26,27,28,29,30,31,32,33,34], patients who have left the department before being seen by a clinician, and excessive patient flow time [35].

Different countries have addressed the problem of ED waiting times in various ways [2,36,37,38]. For instance, in the UK, until recently, EDs were contractually mandated to treat and discharge 95% of patients within four hours [39,40,41,42]. Meeting the QI has been difficult [43,44], and discussions are underway for alternative measures with one such measure requiring time to initial assessment within 15 min of arrival [45,46]. At present, however, EDs worldwide are struggling to meet waiting times [1,36,38,47].

### 1.1. Review of the Literature

Several studies have identified bottlenecks as a contributor to ED problems including increased length of stay (LoS) [23,30,48,49]. One study classified factors underlying the problem of bottlenecks into input, throughput, and output factors [30]. While input factors reflected sources of bottlenecks that were generated in the patient inflow, throughput factors were those that occurred within the ED. Output factors were seen as bottlenecks arising in other parts of the health system that affected the ED. For example, from an input perspective, all patients in the ED must be seen by a doctor within a particular time [50]. However, increased incoming demand can generate bottlenecks as more patients wait to be seen. Moreover, timely throughput is challenging for many EDs [9,48,51]. For instance, specialist consultation, necessary to process complex patients, is a common throughput-related bottleneck [50,52]. An output-specific bottleneck might be discharged patients who are unable to egress the ED due to the non-availability of transportation [53].

Patient processing within an ED can be characterised by complexity. For example, patient care is activity intensive. The patient can be viewed as the primary unit of information, and the clinician interacting with other staff and resources carries out a series of activities by acting upon that information to arrive at certain decisions. If diagnosis and subsequent treatment involve interactions between increased numbers of staff and resources, then this can generate complexity. Moreover, the interactions may be intra-departmental, such as the receptionist notifying a triage nurse about a patient in the waiting area, or inter-departmental, wherein the ED must access other units such as laboratories. Such interactions indicate that an ED, itself a complex system [54] comprised of various physical (e.g., Resuscitation, See and Treat unit, etc.) and non-physical (e.g., care pathways, data flow) sub-systems within its own boundaries; is also part of a system of systems [55], as it relies on other independently functioning sub-systems such as ancillary services, specialty wards, etc., to ensure its performance. While the ED can exercise a degree of control over functions within its boundaries, interactions with other sub-systems can also lead to variations in the ED’s operational performance.

To date, several approaches have been trialled to address ED bottlenecks [18,23,34,35,56,57,58]. Some of these have involved real-world interventions such as doctor-led triage, rapid assessment, fast tracking, streaming, alternative admission options during periods of access block, expansion of nursing scope of practice, the co-location of a primary care clinician in the ED, and Point-of-Care Testing (POCT), which have yielded varying degrees of success [34,56,58]. Other solutions broadly classified under lean, Six Sigma, and continuous quality improvement have also been applied to examine healthcare processes [17,35] including those related to ED bottlenecks [25,59]. For example, SIPOC (Supplier, Input, Process, Output, Customer), a Six Sigma methodology, has been used in processes within the obstetric unit [60]. The tool is useful in terms of gaining a higher-level view of processes comprised within a complex system. However, upon gaining the higher-level view, a detailed process model is needed for a better understanding of the intricacies on the ground. Solutions underpinned by Operations Research/Management (OR/OM) methods, including simulation techniques, have been commonly used to solve bottlenecks in the ED [23,57,61,62,63,64]. For example, Discrete Event Simulation (DES) has been applied to model EDs at an operational level [64,65]. However, an accurate representation of the systems to be modelled is required as an input. The strength of the input relies on the granularity of the data. The best practices recommend that a diagram, such as a flow chart, be used to help understand the structure and function of the system being modelled [65]. In a systematic review of computer simulation applications in the ED in the UK [63], the authors described the patient flow through the following methods: textual, activity list, or flow chart. However, these methods do not provide a sufficient level of detail that entails patient care processes. System Dynamics (SD) is used in complex systems to model the system’s behaviour over time and to understand how the behaviour is impacted by the structure of the system [63,64]. Through the use of stocks and flows, SD provides a macroscopic view of the system [64]. It captures aggregate rather than individual flows and is useful for strategic planning [66]. Petri nets are useful techniques for modelling healthcare systems [33,67,68]. Similar to DES, Petri nets have also been used to analyse the performance of EDs by modelling patient flow and simulating different scenarios to assess improvement in system performance [67]. The dynamic behaviour of processes can be illustrated and visualised [33]. However, it is recommended that the process starts with an understanding of the system flow that is captured using flow charts [67], which again, does not provide the level of detail necessary for modelling patient care processes.

### 1.2. Need for Granular Understanding of ED Process Flow

As the discussion underscores that, addressing the problem of bottlenecks will first entail an accurate understanding of an ED’s processes in terms of the activities that staff carry out and the interactions they have with other roles and resources involving those activities. This can be viewed as, first, acquiring procedural knowledge, which is usually qualitative in nature and involves the processes carried out by clinicians to operationalise care and, next, representing the gathered procedural knowledge to accurately reflect the service system. The resulting representation of the system can then be used as an accurate input model for the simulation.

Obtaining knowledge about a complex system generally requires information at a granular level [69], though this comes at a cost. At present, no guidelines exist about the minimum level of information that must be modelled to address bottlenecks in the ED. Furthermore, there is a growing need for an accurate understanding of throughput issues involving ED process flows as a way to improve waiting times [4,22,70,71].

In terms of modelling process flow, emergency medicine experts recommend applying system modelling techniques. Widely used in manufacturing, industrial engineering, and complex services [72,73,74], system modelling techniques are increasingly used in healthcare settings. Common among these are dataflow diagrams [75,76,77], flowcharting [76,78,79], Value Stream Mapping (VSM) [25,59,80,81,82], and Role Activity Diagram (RAD) [75,83,84,85]. Table 1 provides a comparison of the methods based on their ability to model attributes of the system, focussing on varying levels of granularity. While SIPOC provides a higher-level view of the processes within a complex system, the approaches in the blue highlighted area are useful for process modelling to understand the system at a greater depth, which is also the key focus of this study. Insights gained from the process modelling can then be used towards the development of realistic models as inputs for simulation and analysis.

Healthcare processes are highly complex [59,62]. Plus, providing care to patients is characterised by interactions between the patient, various staff, and resources such as equipment, scales, etc. Needed, therefore, is an approach that can accurately represent these interactions with sufficient detail. As shown in the table, each of the process modelling techniques have certain advantages, though RAD, by comparison, can model granular information such as parallel and collaborative processes, interactions between roles and responsibilities, along with sequential and simultaneous activities. It also allows visually representing intricacies that are characteristic of an ED, such as multi-level interactions, and is, therefore, able to represent activity-rich processes, which is essential for patient care. The use of RAD also provides the opportunity to model intricacies within a complex system, as it requires in-depth knowledge of processes [86].

Therefore, taking these factors into consideration, this paper proposes to model granular procedural information, gathered at a ‘shop floor’ level, involving the activities that ED staff carry out and the interactions therein between staff and resources in order to derive accurate representations of the processes of care in the department to identify factors that lead to bottlenecks and recommend improvement suggestions aimed at reducing the bottlenecks. The rest of the paper is organised as follows: Section 2 provides the plan of the study. Key findings, including information about improvement suggestions, are presented in Section 3. Next, Section 4 discusses the results in terms of their implications on ED processes, and finally, Section 5 provides the conclusion.

**Table 1:** Comparison of commonly used system modelling techniques.

	**Capabilities**	Classify complex system into processes	Process mapping and modelling							
**Methodology**			Show start and end of a process	Show sequential flow and steps in a process	Show sub-processes	Show decision questions & possible outcome	Show simultaneous processes	Show roles performing activities within a process	Show interactions between roles	Predictive system performance
SIPOC [87,88]		Yes	NA							
Data flow diagram [59,60,61]		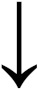	Yes	Yes	No	No	No	No	No	
Value Stream Mapping [23,64,65,66]			Yes	Yes	Yes	No	No	No	No	
Flow chart [21,71,72,73]			Yes	Yes	Yes	Yes	No	No	No	
Role Activity Diagram (proposed in the paper)			Yes	Yes	Yes	Yes	Yes	Yes	Yes	
Simulation approaches *			Process mapping provided through flow chart, textual description or activity list [89]							Yes

Note: SIPOC: Supplier, Input, Process, Output, Customer. * Examples: Discrete Event Simulation, System Dynamics.

## 2. Materials and Methods

Ethical approval for the study was granted by the University of Warwick Biomedical and Scientific Research Ethics Committee (Approval Code: REGO-2015-1715). Site-specific approval was further received from the hospital’s Research and Development office.

### 2.1. Data Collection

The study was carried out in a Level-1 ED located within a 1200 bed teaching hospital in the UK. The site provides physician-led 24-h service 7 days a week with full resuscitation facilities. The ED sees an average of 315 patients daily [87]. Similar to many EDs in the UK, the 4-h QI in the ED was below 95%, showing a decline in performance [87]. Hence, improving ED waiting times is a key priority for the hospital.

A qualitative approach was deemed to be the most appropriate first, as the study attempted to understand processes within an ED by modelling procedural knowledge and due to the exploratory nature of the study. Such an approach can contribute to a greater understanding about the steps of care taken by staff for a particular patient, while also providing insights on factors guiding the decisions around those steps [88]. While quantitative methods such as those discussed in the previous section have been preferred; however, these methods have limitations as they often use simplified process maps of the ED flow based on routinely collected hospital data. A system’s perspective helped guide the data collection, since the ED is comprised of several interlinked units such as Resuscitation, Majors, See and Treat (Minors) clinic, etc. Moreover, an ED is integrally linked to a larger health system and can impact that system while also being affected by it.

From January 2017 through December 2017, a combination of direct observations, discussions with staff, and semi-structured interviews helped to gather the necessary data and information. A sample size of between 20 to 30 respondents is generally accepted for this type of qualitative research [90]; therefore, a sample size of 30 staff was initially planned. Due to the operational nature of the tasks performed, interviewees were skewed toward nursing staff [91]. The nurses who were mainly involved in the day-to-day operations of the units were: (i) the ED Coordinator (EDC), a post held by experienced senior nurses, who had an overarching view of the departmental processes during each shift; and (ii) nurses who worked within the different units of the ED. It was therefore planned that, in addition to the EDC, a minimum of two nurses per unit, i.e., Majors, Resuscitation, and Minors would be interviewed. Participants were chosen from the daily rotas, stratified to include the required staff roles until at least two members of staff from each role had been interviewed. This sampling resulted in a total of 21 staff being invited to participate in the interviews, while feedback from an additional 6 staff was used in the validation process. The participants included two EDCs, two Resuscitation nurses, two triage nurses, two Minor (See and Treat) nurses, two Major leads (ML) who were also senior nurses, two emergency care technicians (ECT), one ED manager, two streaming nurses, an ED physician, a site manager, one ED matron, one charge nurse from Old Persons Assessment and Liaison team, a coordinator, and a nurse from the Clinical Decisions Unit (CDU). The level of experience of the staff interviewed ranged from 15 months (around 1.25 years) to 419 months (34.92 years), with over half of the interviewees having more than 10 years of experience.

Participants were recruited via email and through discussions with relevant managers. All participants received information about the study prior to the interviews. With written consent, the interviews were audio-recorded. Nineteen staff consented to have their interviews recorded, and the responses of the remaining two participants were documented on paper. The interviews focused on gathering in-depth information on activities of care that staff carried out, shared roles, responsibilities, interactions with other staff, decisions made along the patient flow, simultaneous tasks performed, along with resources needed to carry out the activities, including ancillary services, databases, etc. Open-ended themes revolved around challenges faced while providing care, for example, processing complex symptoms, the need for timely information, as well as interviewees’ perspectives on improving ED processes. The interviews were transcribed by one of the co-authors (MA). The transcripts formed the raw data for analysis in the study.

### 2.2. Analysis

An iterative process was taken to develop the RADs. First, using a content analysis approach, all terms relevant to the RADs were systematically extracted from the transcribed scripts including roles, activities, interactions between roles and units, resources, and decision questions along the patient flow. The extracted terms were then used as input into Microsoft Visio to construct the RADs. The specific steps to derive the RADs can be found in earlier publications [75,76]. The resulting RADs generated detailed process maps comprised of activities that were carried out by staff in the department. Process maps are commonly used tools in business and industry to support the understanding of complex systems for quality improvement [92]. Next, a two-step procedure was followed to verify and validate the RAD-based process maps. For verification, first, the RADs were cross-checked against the transcripts. Next, the initial RADs were shown to the interviewees, as they had detailed knowledge of the ED’s processes. The input received was then used to update the RADs. Following this, as validation, the diagrams were presented to staff who were not involved in the interviews, and their input was consolidated after discussion. The final RADs provided an in-depth view of the process models in the ED. We also had communication with an ED Senior Charge nurse, the role centrally responsible for ensuring the department’s functions, in the summer of 2021 regarding the ED’s processes. Based on the discussions, processes related to the focus of this paper have remained unchanged. The RAD notations are presented in Appendix A.

## 3. Results

### 3.1. General Protocol Followed in the ED

**Figure 1:**
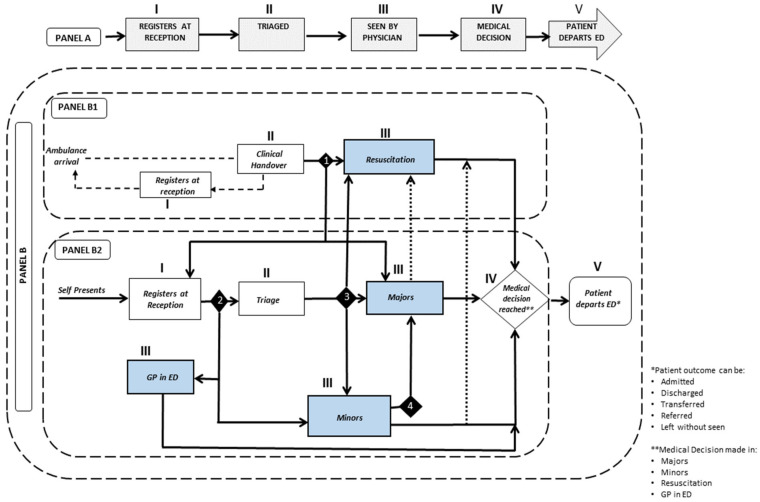
Flowchart of emergency department patient flow. Note: GP-general practitioner (primary care physician).

Figure 1 illustrates an ED patient flow divided into two panels. Panel A shows a generic flow of the broad steps, which represents the common protocol followed in an ED wherein, upon arrival, a patient registers at reception (I) and is then triaged (II). Next, the patient is assessed by an ED physician (III), which can also involve a re-evaluation [46], leading to a medical decision (IV) and subsequent discharge (V) [93].

Panel B, developed using information culled from the interviews, provided a more detailed portrayal of the ED’s patient flow based on the mode of arrival. Four diamond shaped decision points (DP), marked 1–4 in the figure, were also included, which represent changes in patient flow depending upon decisions made by staff. Additionally, five processes, noted as I–V, coincided with the five steps shown in Panel A. In measuring total time spent in the ED, the clock started at patient handover for ambulance arrivals, whereas for self-presenters, from registration at reception. The clock stopped at patient discharge from the department.

Panel B1 illustrates the main steps that are carried out for patients who are ambulance conveyances. Upon arrival, a clinical handover of the patient is conducted by paramedics to the ED staff. As paramedics egress the department, they check-in with the receptionist and provide a copy of the patient report form (PRF). Meanwhile, staff conduct a rapid assessment of the patient to reach a decision (DP1) on whether to send the patient to Resuscitation (resus); Majors, the unit within the ED that sees serious and complex illnesses and injuries; or to the regular waiting area if the patient is deemed suitable to wait.

Panel B2 depicts the self-referral route, where, upon arrival, patients register at reception. A streaming nurse conducts a visual assessment of all patients and directs them to the appropriate area (DP2), which can either be a general practice (GP) clinic, located in the ED, for patients meeting the GP care criteria; Minors (See and Treat) unit for non-life-threatening conditions; or Triage, where a nurse sees the patient while simultaneously ensuring that all patients waiting are directed to various units within the ED (DP3). Occasionally, due to emergent conditions [89,94], patients sent to Minors could be redirected to Majors [DP4]. While deteriorating patients in Majors or Minors might necessitate transfer to Resus, such changes were infrequent at the study site and are therefore shown with dotted lines. A patient is discharged upon treatment completion. The 4-h QI was measured from time of arrival to departure from the ED.

Panel B2 began to shed light on the non-linear nature of the ED’s patient flow. The interviews further revealed that the main flow into the GP-in-ED and Minors clinics comprised self-presenters. The flow into Resuscitation was mostly by ambulance arrivals, whereas Majors received patients from both routes, i.e., self-presenters and ambulance conveyances. Furthermore, emergent conditions could also result in entry to Majors from other areas, as shown in Figure 1. These factors signified varied levels of care complexity in Majors. The interviews additionally indicated Majors to be the most crowded area in the ED, a finding reported elsewhere [95]. Furthermore, a snapshot of anonymised routinely collected data, shown in Table 2, involving adult patients extracted from the hospital’s electronic database for the year 2018, showed that, while a majority of the patients were seen in the Minors unit (52.54%), most of the patients who violated the UK NHS mandated 4-h waiting time quality indicator were seen in Majors. This accounted for 63.17% of violators, even though they made up a little over 36% of the patient visits, raising further concerns about crowding in Majors. Hence, a decision was made to map the process flow in Majors.

### 3.2. Granular Process Modelling of Care in the Majors Unit

**Figure 2:**
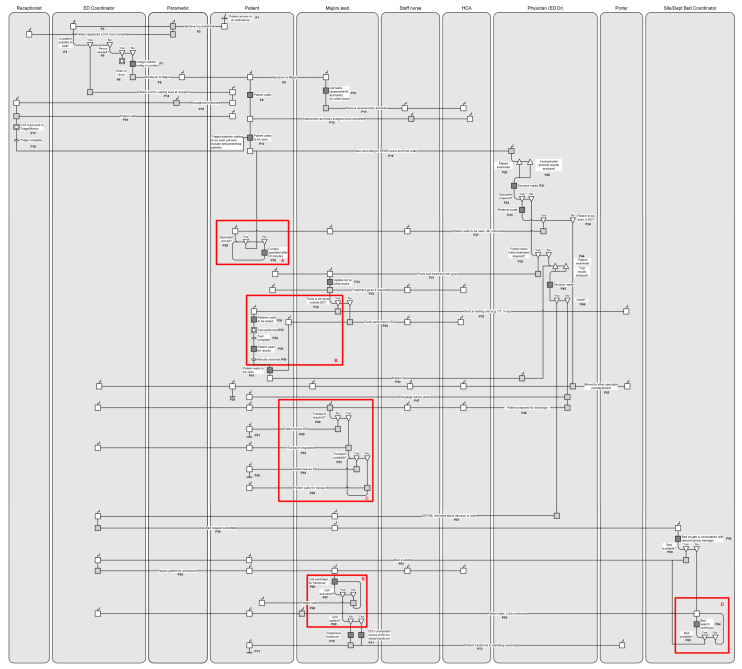
RAD of a patient’s journey through the Majors unit. Note: Phys: Physician; SEWS: Standardised Early Warning Score; CT: Computerised Tomography, CDU: Clinical Decision Unit; Resus: Resuscitation area; A: Awaiting specialist team; B: Undertaking a test outside ED; C: Awaiting transport; D: Bed search; E: Handover to admitting ward/department.

Figure 2 shows the RAD-based process map depicting activities within Majors starting with ambulance arrival. The figure parses out the activities surrounding a single patient’s care journey, decisions made along the care flow, and the impact of the decisions on that journey. The figure is presented in a level of detail to fully communicate the intricacies that arose as a result of the decision making and the snowballing effect the decisions had on staff activities. In light of the current discussion underscoring the need for more informed ED waiting time measures, a detailed depiction of the process flow within Majors seemed especially necessary. A total of nine staff, located within and outside Majors, had interacted to provide the necessary care. As the chief source of information driving the process, the patient was represented as a role. Depending upon the decisions made, processing a single patient in Majors involved 12 steps comprised of 73 processes (denoted with the letter P). While the processes are routinely carried out by staff in Majors, these processes have never been documented. Therefore, to facilitate a better understanding of the unit’s process flow, the steps are briefly summarised below.

*Handover* (P1–4): Upon arrival, paramedics hand the patient over to the ED Coordinator (EDC) who determines if the patient is suitable to wait. As paramedics egress the ED, they provide a copy of the patient report form to the receptionist, who uses the information to generate an ED CAS (casualty) card.

*Resus/Majors* (P5–8): A patient who is not suitable to wait is seen in Resus. Those with serious but not life-threatening illnesses are assigned a cubicle or trolley in Majors and handed over to the Majors Lead (ML).

*Assessments and tests* (P9–13): While the patient waits, the ML lists, on a centrally located whiteboard, all assessments and tests that the patient must undergo and assigns these to an HCA and staff nurse.

*Triaging/Minors* (P14–18): Patients deemed suitable to wait are either sent to the waiting area to be triaged or seen in Minors. Meanwhile, the receptionist electronically sends the CAS to the appropriate unit, shown as an encapsulated process.

*Seen by ED Physician* (P19–26): Based on a patient’s severity score and time order of arrival, a physician sees the patient, reviews any tests, decides if a specialist is needed, and makes the referral. If the patient is to be seen by specialist outside the ED, then the patient is moved to that area. This concludes the patient’s ED LoS.

*Seen by Specialist* (P27–30): A reminder to the specialist is made by the ML after 30 min if the specialist has not arrived. If, upon assessment, a different specialist is needed, then the chain of referrals continues until the patient is seen by an appropriate specialist, which can lead to requiring more tests or treatments.

*Additional tests/treatment* (P31–37): The ML ensures that all tests and treatments have been performed by the HCA and staff nurse. If tests are to be performed outside of the ED (scans and x-rays), then a porter transports the patient to the test area for a test to be performed, which is shown as an encapsulated process in Figure 2.

*Medical decision* (P38–46): The patient continues to wait until test results have arrived. This also includes patients who had tests in ED, such as a point-of-care test (POCT). Whilst being seen by a physician, test results are analysed, and a medical decision is made about whether the patient is to be admitted.

*Discharge* (P47–48): Patients not requiring admission are given discharge advice by the physician. Discharged patients needing medication will have it dispensed from the ED, or if not in stock, then it is requested from the hospital’s pharmacy.

*Departure* (P49–56): Departure from the ED denotes the end of the care process, and the clock monitoring LoS is stopped. Where patients require transportation to exit the ED, staff must make such arrangements.

*Admission/Bed search* (P57–65): For a patient requiring admission, a request for a bed is raised by the EDC to the appropriate site manager, which prompts searching for a bed in the specialty area. Simultaneously, the patient is prepared for admission. However, if no bed is available, then the patient continues to wait in the ED and is monitored by ED staff.

*Handover to admitting area* (P66–73): Once a bed becomes available, a handover call is placed to the receiving area by the ML until it is answered. For CDU patients, the unit’s coordinator arrives in ED for a verbal handover. Next, with the help of a porter, the patient is moved to the receiving ward and the clock overseeing the ED LoS is stopped. Concurrently, all notes are scanned into the electronic patient records system as part of the patient discharge process.

A review of the processes revealed five instances of bottlenecks in Majors. Marked with red boxes in Figure 2, these were awaiting specialist (A); tests outside the ED (B); discharged patients waiting for transportation (C); inpatient bed search (D); and handover between ED and inpatient ward/department (E). Since these bottlenecks are common problems in EDs [36,48,96,97,98], the extant literature was first scanned for improvement suggestions that are currently recommended to deal with these bottlenecks. The suggestions, shown in Table 3, can be classified under four broad groupings, namely, re-allocating resources to the bottleneck area; moving tests upstream; creating buffer zones to ensure timely discharge from the ED; and better handling of data and information.

**Figure 3:**
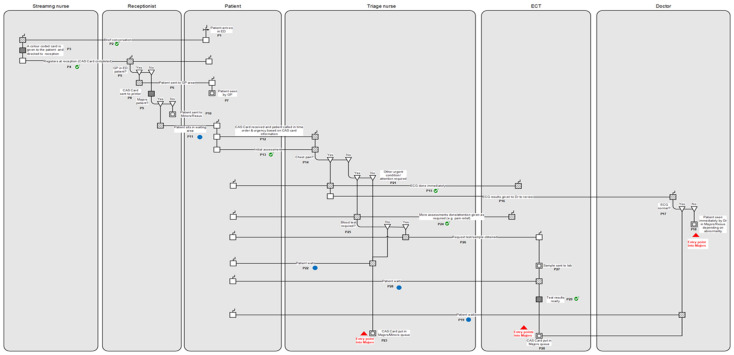
RAD of information collection points prior to entry into Majors (self-presenting patients). Note: Self-presenting patients are triaged and sent to the Majors unit if appropriate.

To explore improvements aimed at addressing the identified bottlenecks (Figure 2), further mapping was conducted to trace the ED care journeys of patients prior to their entry into the Majors unit. Figure 2 detailed the journeys for those who had arrived by ambulance; hence, the mapping at this juncture focused on self-presenting patients. Figure 3 illustrates the journey for this group up to the point of their entry into Majors. A total of five roles, namely, streaming nurse, receptionist, triage nurse, emergency care technician (ECT), and doctor, had interacted to provide care. Upon arrival, a streaming nurse conducts a quick assessment of all patients and indicates the unit for them to register at reception. Patients suitable for GP care are directed to the GP-in-ED clinic. Those who do not meet the Majors criteria are sent to Minors or, if necessary, Resuscitation. After registering a patient, the receptionist generates and sends the CAS card, containing preliminary patient information, to the relevant area. All remaining patients are triaged and assigned a SEWS. An immediate ECG is conducted for patients experiencing chest pain and the results are provided to the doctor while the patient is sent to Resuscitation or Majors based on urgency, thereby denoting entry into Majors (Entry point 1). Patients with normal ECGs must wait to be seen while their CAS card is placed in the Majors queue, which also represents entry into Majors (Entry point 2). Non-chest pain cases are assessed for other urgencies, and if suitable to wait, their CAS cards are also put in the Majors queue, indicating entry into the unit (Entry point 3). For patients with urgent conditions requiring further assessment, such as a blood test, an ECT obtains the sample and sends it to the lab. If such tests are not needed, then the patient waits to be seen and the CAS Card is put in the queue.

### 3.3. Suggestions to Address the Identified Bottlenecks in Majors Derived from Process Mapping

A closer examination of Figure 2 and Figure 3 generated insights that could help to manage and potentially reduce the occurrence of the identified bottlenecks via three means, namely, using precedence information to reduce repeat tests; developing an informed alert system; and addressing variation, especially with respect to time-based QI.

#### 3.3.1. Use of Precedence Information to Reduce Repeat Tests

Performing tests is a time-consuming interaction. However, the process mapping also indicated a flow of information with staff having access to certain data about patient health, collected prior to Majors entry, which could be used to address some of the bottlenecks. For instance, all patient arrivals via ambulance are accompanied by the Patient Report Form (PRF) [105,106] (or its electronic equivalent, the ePRF), which is generated by paramedics [106]. A copy of the PRF is provided to ED staff at handover [106] [Figure 2; P2]. Additionally, the CAS card [107,108] is generated for all patients at registration and information is recorded in it along the patient flow. Table 4 shows a set of variables extracted from the PRF and CAS that could be used to process certain patients efficiently, thereby reducing instances of bottlenecks. The CAS-based variables in the table are collected at triage, which represents an early point in the patient flow.

Patients with chest pain often experience prolonged waiting times, as diagnosis can require a blood test [109]. Here, results from pre-hospital blood tests conducted by paramedics can speed up decision making in the ED, thereby potentially reducing patient waiting times [110,111]. Imaging is another area where bottlenecks have been reported [49]. However, the use of point-of-care ultrasound imaging can be promising [112]. Hence, where available, EDs can consider using pre-hospital ultrasound results, which could then reduce some instances of bottlenecks [113]. Delays in diagnosing cerebrovascular events in ED such as stroke are equally problematic [114]. However, the recognition of stroke by paramedics is shown to have a high rate of agreement with ED assessment [115], and many EDs use the results to fast-track patients to the hyper-acute unit [115]. This could be further explored with respect to Majors’ patients. Overall, health history, vital signs, observations, and presenting complaint (source: PRF and CAS) are regularly used by EDs to treat patients. These parameters can also help to see if requests for certain tests, especially those that need to be conducted outside the ED, can be submitted in advance of the patient being seen, which could lead to better management of bottlenecks.

#### 3.3.2. Developing an Informed Alert System to Alleviate Waiting Time Pressure

The mapping also provided clues on alerting staff at key points of the patient’s ED care journey that could potentially reduce some of the bottlenecks. For example, some patients access EDs frequently. These include the elderly, those with complex clinical and psychosocial conditions, and patients with past hospital admissions [116]. Although a proportion of such patients will require care at the time of their visit, it will be important to assess if inpatient admission is necessary. For this, examining processes earlier in the patient flow can be useful. For example, at P8 (Figure 2), less severe cases arriving by ambulance were handed over to Majors, and at P10, the ML listed tests needed for these patients. The ML also has access to the CAS and PRF, which contain information on demography, previous attendances, health and medication history, risk of falls, and mental health. Hence, processes at P10 could be enhanced by incorporating validated scales [117], for example, to assess for frailty in the ED. A lower score could lead to alerting the discharge team, which can then put in place an appropriate package of care to facilitate safe and direct discharge from the ED.

At the hospital, some patients were unable to exit the ED upon discharge due to lack of transportation, a problem that has been reported through other studies as well [53,118]. As a result, patients ended up boarding while ED staff had to spend time arranging for transport. This can divert staff time necessary to process other patients and add to crowding [53]. The need for transportation is recorded on the CAS card when a discharge decision has been made (Figure 2: P49). However, the mapping showed that this information can be collected early in the process flow, i.e., triage (Figure 2: P12, Figure 3: P13), which can then be used to send an early alert to the ED discharge team for planning purposes. Where a patient ends up getting admitted, the transport arrangement could be used for another patient. In addition, age, vital signs, health history, and falls risk can also provide indications about whether a patient might encounter delays exiting the ED. Having such information early in the patient flow can help with better discharge planning and reduce instances of transportation-related bottlenecks.

Awaiting specialist input was also seen to prolong ED LoS. An important consideration when requesting specialist input is the time needed by a specialist to respond to a referral. In the ED, all patients were assessed and assigned to be seen in the appropriate unit based on the severity of the presentation (Figure 2: P4, Figure 3: P3). Generally, those with moderate levels of severity were seen in Majors, with a proportion of such patients requiring specialty input. In terms of reducing specialist-related bottlenecks, first, an interaction with an advanced care practitioner (ACP) can be implemented at triage, as ACPs are trained to support decision making involving undifferentiated presentations [119]. Furthermore, in cases where an ACP identifies a specialty, an alert could be sent to that specialty, to help with planning in that area. Then, following patient review by the ED physician, depending upon the degree of agreement between the physician and the ACP’s input about the specialty, the alert could either be followed through or used towards the next applicable patient. While ACP input at triage might not prevent every instance of specialty-related bottleneck, it can potentially reduce a proportion of such bottlenecks and help to speed up ED patients processing.

#### 3.3.3. Understanding Variation in the Context of Time-Based Quality Indicator in ED

Timely patient processing is singularly important for an ED. However, EDs represent fast-paced environments characterised by complexity at all levels, i.e., patients, providers, decision making [54], and resources. A majority of patients seeking care in an ED arrive with unclear symptoms, and clinicians working with partial information endeavour to arrive at a decision about a condition. If decision making requires further information, an instance that denotes complexity [54], then the clinician requests additional information such as tests and opinions. From an operational perspective, accessing information to process patients in the ED can be realised through activities that are carried out either within the department or through interactions with units located outside of the ED, the latter commonly self-organised around their own set of rules, for example, laboratories. In addition to generating bottlenecks in the ED, as was seen in this study, such interactions can also introduce variation in care [92].

While some variation emanates from uncertainty related to a patient’s condition and response to treatment, other sources of variation might be operational, i.e., non-clinical, in nature. The inpatient admission process served as an example, wherein even when a patient was admitted to an inpatient ward, the handover to the ward could not be completed due to the non-availability of ward staff, which then precipitated a prolonged ED LoS. Timely handover is crucial for ensuring appropriate care and any disruption can have a negative effect on the ED process flows, as staff must meet incoming demand while diverting time to admitted patients.

Reducing bottlenecks in EDs will require better planning, and this necessitates understanding how variation, both due to clinical as well as non-clinical factors, affects ED process flows. To that end, collecting and modelling granular information is an essential first step towards understanding the variation. At present, simulation studies are often conducted to help EDs reduce bottlenecks [37,62,120]. However, improvement suggestions from these studies can be made stronger through the use of realistic models as inputs to the simulation [121]. Results can lead to developing informed improvement suggestions with respect to reducing ED bottlenecks.

## 4. Discussion

By applying a systematic approach, role activity diagram-based process mapping, this paper attempted to model granular information involving care processes in a Majors unit, located within a Level-1 ED, to understand how activities carried out by staff and interactions therein impact bottlenecks and waiting times. Results have implications on strategies necessary to help EDs meet waiting time indicators and quality of care.

To our knowledge, this is the first process mapping to illustrate the flow of work in Majors involving a Level-1 ED. Numerous efforts are underway to help EDs improve waiting times. It will be crucial, however, that such efforts are underpinned by an in-depth understanding of the routine complexity that staff must handle on a daily basis when processing patients. Evidence indicates that Majors is associated with crowding, and this might be due to the complex nature of the care provided in the unit. This then makes having a granular view of the unit’s process flow a necessity for identifying factors that contribute to bottlenecks. Plus, a detailed view can aid in understanding processes surrounding a bottleneck, which can be valuable for developing solutions to tackle waiting time problems.

All EDs follow a broad set of steps to process patients (Figure 1). However, emergent factors involving patient condition as well as the system itself, for example, lack of resources, can lead to variations in operationalising the steps and generate tremendous complexities, influencing decision making downstream. The use of RAD, as a process mapping tool, allowed illustrating these features by uncovering how variations in the steps can add a sheer number of activities, which then impact staff workload and LoS. Indeed, the RAD-based process maps revealed several instances where clinical decision making was affected by non-clinical (logistics) factors and in turn, prolonged ED LoS. For example, executing a single discrete non-clinical task such as finding a bed for just one patient translated to multiple roles carrying out multiple sequential as well as parallel activities based on multi-level communications using multiple sources of information and, at times, having to repeat these processes. A similar example involved transportation arrangements by ED staff at patient discharge, which also resulted in prolonging LoS. Often, these activities carried out by staff remain unseen, though they routinely occur alongside the obvious steps. These unseen activities have real implications on ED waiting times.

It is worth noting that treatment in an ED can necessitate the activation of care pathways. This takes on added importance in the case of complex conditions, the type of care that was associated with Majors, making time criticality especially vital since patients must be quickly and accurately diagnosed and placed on correct pathways. Generally, care pathways have been shown to enable timely assessments [122], integrate evidence-based care, and improve the consensus on treatment plans [123]. Nonetheless, evidence suggests that they may be less effective for variable patient journeys [123]. This is problematic since the care flow is not always linear and variations from the pathway do occur either due to patient characteristics or as a result of non-clinical factors such as site-specific processes [92]. Indeed, it is recommended that care pathways be developed, taking into account clinical and system-level elements to ensure meeting care requirements [124]. To that end, the application of RAD holds valuable clues when it comes to the development of care pathways and their implementation. Understandably, care pathways are developed by placing clinical considerations in precedence. However, as was seen in this study, clinical factors often interact with the non-clinical ones within the service system and can result in variations in care processes. Problems that arise when operationalising care pathways, whether in the context of an ED or more generally, can be potentially prevented if their development routinely incorporates both clinical and system-level realities.

Clinical decision making in an ED is frequently dependent on resources that are located outside of the department [2,36,102], as was also noted through the process mapping. While units that house a particular resource might prioritise ED requests [2,102], this may not always be the case, meaning that an ED might have little control over these resources. In this study, most of the bottlenecks arose as a result of the ED having to wait for other units to provide either the necessary information or resources to complete patient processing. While EDs will continue to depend on other parts of the hospital to treat and discharge patients in a timely manner, the process mapping also revealed opportunities for the ED to take a prospective approach concerning planning its access to external resources. For example, Figure 3 showed that key pieces of information are collected at several stages, check marked on the figure, along the care flow before the patient even enters Majors (P18, P20, and P23). Such information can serve as possible indicators for certain resources and early alerts could be sent to the relevant units. This can also provide preliminary indications on capacity planning for the department based on incoming demand. Moreover, the process maps also brought to light several instances where the patient was waiting, some of which, while necessary, nonetheless adds up to the overall LoS. However, scrutinising the maps also indicated that certain relevant care-specific information is collected at early points on the patient’s care journey and is available to staff. Hence, efficient use of the information can potentially help to reduce some of the bottlenecks and reduce patient LoS.

We believe that granular information modelling can also provide important clues towards the development of ED waiting time indicators. The mapping highlighted processes, sub-processes and associated activities that have implications on care quality, patient safety and workload. The goal is to reduce the bottlenecks and prevent these so that they do not affect waiting times. While process mapping alone will not be able to provide all the elements necessary to analyse the ED as a system, granular information can help to derive a better understanding of the complexities that are associated with the system. Results can be used to develop accurate representations of the system, which can then be tested with data to examine factors necessary for developing waiting time indicators.

This study had a number of limitations. First, it was a single-site study, albeit one of the largest acute hospitals in the UK. Additionally, there was a bias towards the clinical view, as the sample mainly entailed clinical staff; although, for the purposes of this study, it was important to gain their perspectives. Moreover, as process flows in the ED are constantly reviewed, the information captured during the interviews may have changed; though, in this regard, the RADs can be of benefit to model “as is” and “to be” processes.

In addition, the activities performed by a role, decisions made, and interactions between roles were captured without matching the roles with the specific duration of a particular resource’s utilisation, providing an important avenue for future research. As this study explored understanding on the ground procedural information, it lacks the use of quantitative data to test some of the improvement suggestions.

The data were collected pre-pandemic, the onset of which required the introduction of hot and cold areas in the ED. Similar to other EDs, there was a reduction in attendance at the beginning of the pandemic [125,126]; however, the problems that existed pre-pandemic are still prevalent, resulting in poor performance of waiting time targets [125], hence making the focus of this paper current.

## 5. Conclusions

A time-based QI overseeing care in EDs has a wide interest [1,2,3,4,5,6,7,8]. While output factors are an important part in the waiting time equation due to the declining bed base, coupled with a reduction of activities in many areas of the health system during out of hours and weekends [2,36], solutions focused on input and throughput factors remain equally important. The lessons learned from the RAD-based process mapping have importance from practical and managerial perspectives in terms of managing waiting times in the ED. The specific factors driving ED crowding vary based on the local context of a hospital, and the success of any process improvement initiative is highly dependent on the process of identifying the causes and, subsequently, addressing them sustainably. Improvement approaches can only be successfully adopted if these are contextualised to a particular hospital [18]. This underscores the need to first develop accurate representations of an ED’s processes by modelling information about these processes at the granular level. For example, the developed RADs can be understood by ED managers and clinicians, who have in-depth knowledge about the processes but need visual tools to help identify problematic areas and provide clues for addressing them, which was also the case in the current study. Indeed, during the verification and validation process, ED staff were intrigued by the sheer number of processes that they performed, something that they saw documented in detail for the first time through the RADs.

Moreover, the process maps showed points along the patient journey where important information was being gathered on care specifics, especially at the very onset of the patient’s ED care journey. This can help staff to explore how measures can be put in place early on for better management and planning to reduce the bottlenecks and perhaps even prevent their development. Indeed, this also provides an opportunity for the hospital, working with its information governance and ICT units, to integrate the sources of information points into the ED’s existing Electronic Health Records Systems and generate a checklist that can be made available to staff. Such a checklist can help to improve and monitor care delivery while also facilitating a standardisation of ED processes.

Resources are limited and ED clinicians and managers need to know which units in the department to focus attention on when embarking on process improvement tasks. At the site, ED staff are being prompted about the need to focus on challenging units within the department to yield benefits that will affect the whole department. The process mapping in this study identified Majors as a unit that staff can focus on in a targeted manner and use the developed RADs for rapid analysis of problem areas. The results can then be integrated with a whole system approach to help the ED improve waiting time performance.

Since reducing the bottlenecks will require developing and testing process improvement strategies, as the next step, our research will incorporate quantitative data into the learnings from this study to examine waiting times. It will entail the integration of quantitative analysis to merge lessons from both the quantitative and qualitative sides to build a DES model for testing scenarios based on the suggested solutions. This study illustrates the importance of granularity for obtaining real-world information from the ‘shop floor’ as an important first step in examining complex systems. Routinely collected data do not contain all the data points captured through the RADs. This provides the opportunity to complement the DES model with the granular process map, along with resource utilisation information, to support discussions on the real-life implementation of solutions.

## Figures and Tables

**Table 2:** Number of patients and length of stay per unit.

ED Unit	LoS < 4 h		LoS ≥ 4 h		Total n (%)
	n (%)	Mean (SD)	n (%)	Mean (SD)	
GP-in-ED	4617(5.25%)	129 (53)	122 (0.53%)	318 (78)	4739 (4.28%)
Minors	53,300 (60.84%)	144 (57)	4832(20.98%)	342 (92)	58,132 (52.54%)
Majors	25,448 (29.05%)	184 (51)	14,551 (63.17%)	433 (171)	39,999 (36.15%)
Resuscitation	4248 (4.85%)	185 (53)	3530 (15.32%)	447 (160)	7778 (7.03%)
Grand Total	87,613		23,035		110,648

**Table 3:** Recommendations to manage common bottlenecks in the ED compiled from the literature.

Bottlenecks Identified	Improvement Suggestions to Alleviate the Bottleneck	Trade-Off	
		Positive	Negative
Reallocate resources to bottleneck area			
Awaiting specialist input	Clinicians from inpatient specialties can be freed up from elective and non-clinical activities to facilitate quicker responses to the ED. Automated reminders [99,100] can be sent to the specialties in addition to the ability for specialties to review results of investigations through electronic means, hence, only needing to visit the ED in person if a physical evaluation is required [101].	Quicker patient processing in the ED, thus helping to meet waiting times.	Inpatients may be affected by having to wait longer to be seen by specialists.
Move tests upstream			
B. Tests outside ED	Facilitate front loading tests [36]. Service level agreements can ensure faster turnarounds for tests by prioritising ED requests and POCT [2,102]. There are agreements at the site for a maximum 2-h turnaround for blood tests; this can be explored for other tests.	Quicker patient processing in ED, thus helping to meet waiting times.	Resources needed to meet service level agreements. Non-ED patients might have to wait longer for tests.
Create buffer zone			
C. Awaiting transportation	Use a discharge lounge to facilitate quicker discharges from the ED [103,104].	Timely patient discharge from ED. Fewer problems with boarding	Increased use of the discharge lounge will require extra resources.
D. Bed search	Evidence on inpatient boarding suggests that admitted patients awaiting a bed can wait in the inpatient ward [2,45,102].	Can be initiated early in the patient stay. Reduce boarding. Free up staff time to see other patients.	Bed availability could be dependent on factors that are outside the control of the hospital.
Better data and information handling			
E. Handover to admitting ward	Integrated electronic notes and handover reports can expedite processes [102], thus reducing the need for verbal handover. Better documentation of admission processes could reduce duplication [2].	Patients will egress the ED on time.	The cost involved in implementing an integrated electronic system.

Note: POCT: Point-of-care testing.

**Table 4:** Information available to staff prior to patient entry into Majors.

Variables of Interest	Patient Report Form ^1^	Casualty Card ^2^
Demography (age, gender)	x	x
Incident date and time	x	-
Date and time of arrival	x	x
Date of birth	x	x
Specialty	-	x
Source of referral	-	x
Number of previous attendances	-	x
GP detail	x	x
Patient transported with an alert	x	x
Risk of fall risks	-	x
Health history	x	x
Vital signs and observations	x	x
Pre-hospital blood test	x	-
Pre-hospital ultrasound	x	-
Cardiac health and ECG reading	x	-
Cerebrovascular events, such as suspected stroke	x	-
Any history of medication	x	x
Mental health among others	x	-
Presenting complaints	-	x
Signs of infection	-	x

Note: ^1^ Source: Paramedics; ^2^ Source: ED.

## Data Availability

Data presented in this study are available on request from the corresponding author. The data are not publicly available due to ethical and privacy guidelines.

## Appendix A

**Table A1:** A description of RAD concepts and corresponding graphical representation [76,127].

No.	RAD Concept	General Description	Example (from Figure 2)	Graphical Notation
1.	Role	A role performs a set of activities to achieve a particular goal. A role can be an individual, a group of people, or equipment.	ED coordinator, paramedic, and staff nurse.	
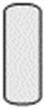
2.	Activity	A unit of work performed by a role is an activity.	List jobs on board, call ward/dept.	
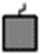
3.	Interaction	Interaction represents a collaboration between roles to achieve the objective of the process. The role with the shaded box is the driver or the interaction, and the plain box is the receiver. There can be multiple drivers and receivers for an interaction.	Handover by a paramedic to ED coordinator, Major leads discuss tests and assessments with staff nurse and HCA.	
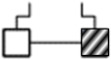
4.	Case Refinement	A case refinement represents a decision question and the possible outcomes.	Decision question: Bed available? Outcome: Yes or No.	
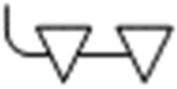
5.	Part refinement	The part refinement symbol represents activities done simultaneously by a role.	Patient examined, assessments and test results analysed simultaneously.	
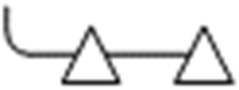
6.	Trigger	A trigger represents an event that starts the activity thread.	A patient arrives in an ambulance.	
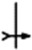
7.	Encapsulated process	An encapsulated process symbol represents a subprocess on the main diagram. The subprocess is then expanded on a separate diagram.	Tests outside ED.	
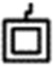
8.	Loop	A loop symbol is used to represents a part of the process that repeats itself	Is transport available? If ‘no’ then patient waits, and the question is repeated until the answer is ‘yes’.	
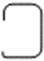
9.	Stop	The stop symbol marks the end of a process by ending a thread.	After the patient leaves the ED, the thread ends.	
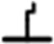
10.	State	The state symbol is used to describe what is true before or after an action.	Test complete.	
